# Are doping tests in sports trustworthy? Athletes suffer from insufficiently defined criteria for doping tests

**DOI:** 10.15252/embr.202154431

**Published:** 2022-02-14

**Authors:** Jon Nissen‐Meyer, Tore Skotland, Erik Boye

**Affiliations:** ^1^ Department of Biosciences University of Oslo Oslo Norway; ^2^ Department of Molecular Cell Biology Institute for Cancer Research Oslo University Hospital Oslo Norway; ^3^ Department of Radiation Biology Institute for Cancer Research Oslo University Hospital Oslo Norway

**Keywords:** Economics, Law & Politics, Methods & Resources

## Abstract

The lack of clearly defined criteria for doping tests carries a great risk of punishing innocent athletes and undermines the fight against doping in international sports.

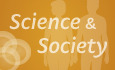

The World Anti‐Doping Agency (WADA) uses analytic, science‐based methods to detect doping, but it does not always adhere to scientific principles when it evaluates the results from their tests. The criteria for determining whether a sample is positive for an illegal substance often appear to be ambiguous with the risk of rendering evaluations subjective. Statements from WADA laboratories such as “you need to be an expert to clearly identify it” and “we know it when we see it” indicate such subjectivity. Subjective evaluations are troublesome because they erode the trust in WADA’s fight against doping, and have potentially dramatic consequences for athletes.

## Non‐threshold tests

This subjectivity affects the majority of anti‐doping tests, namely the detection of so‐called non‐threshold substances. These tests lack objective and quantifiable decision limits that undisputedly resolve whether test results should be interpreted as positive or negative. According to WADA’s specifications (td2019mrpl_eng.pdf (wada‐ama.org)), such tests may be judged positive regardless of the amount of the substance in question, as long as it can be detected with reasonable certainty. Moreover, some laboratories are capable of detecting lower concentrations of prohibited substances than other laboratories, simply because the laboratories may use different equipment and/or test methods (td2019mrpl_eng.pdf (wada‐ama.org)). Whether or not an athlete tests positive will therefore vary from laboratory to laboratory. The call for defining unambiguous criteria for doping analyses and their proper validation has been out for decades (Berry, 2008), but apparently with little response from WADA.

The decision limit affects the specificity and sensitivity of the test. A low limit decreases the specificity and thus increases the frequency of false positives, whereas a high limit decreases the sensitivity and increases the likelihood of false negatives. Consequently, a lack of decision limits precludes an assessment of either specificity or sensitivity. Moreover, it prevents objective test evaluation because decisions become arbitrary.

Whether or not an athlete tests positive will therefore vary from laboratory to laboratory.

The case against the German biathlete Evi Sachenbacher‐Stehle is a sad and telling example of what can happen in the absence of a clear decision limit. Sachenbacher‐Stehle tested positive for a tiny amount of methylhexanamine during the 2014 Winter Olympics in Sochi, Russia. The head of the Sochi laboratory, Grigorij Rodchenkov, later admitted in his book that he normally would not have pursued the case, given the low level of the drug (Rodchenkov, 2020). However, the laboratory needed some positive results, partly because Russian athletes with positive tests were not reported, so he called this test as positive. Sachenbacher‐Stehle was initially suspended for two years for what was likely due to consumption of contaminated tea (https://fasterskier.com/2014/07/sachenbacher‐stehle‐case‐raises‐questions‐about‐supplement‐use‐and‐safety/). Rodchenkov was apparently free to choose whether Sachenbacher‐Stehle should be considered a doper or not and whatever he decided was in accordance with the rules.

## Subjective decisions

This example illustrates how the lack of well‐defined decision limits allows subjective decisions. When athletes challenge the decision, arbitrators on a hearing panel normally show full confidence in the laboratory staff’s opinion, which makes it nearly impossible for athletes to prove their innocence. Moreover, athletes may neither have the financial means nor access to legal advice and scientific expertise to challenge the verdict. The case against the Irish student and amateur sprinter Steven Colvert provides another illustrative example (Nissen‐Meyer *et al*, 2016, 2019; Boye *et al*, 2017). In his case, gel electrophoresis was used to detect recombinant erythropoietin (rEPO) in his urine. This technique generates results that are less quantitative – and therefore more prone to subjective evaluation – than the tests for most other drugs. The WADA‐accredited laboratory in Cologne, Germany, interpreted a slight tailing or spreading above Colvert’s normal endogenous EPO as being caused by rEPO. This tailing was, however, not easy to see and not much different from that observed in parallel lanes containing the urine of athletes devoid of rEPO. When Colvert’s defence raised doubts about the ambiguous test results, representatives from two WADA‐laboratories stated “…a sample which has such low doses of recombinant EPO, you need to be expert to clearly identify it…”. This is not a scientific argument, but the arbitrators nevertheless found Colvert guilty of doping, a verdict which has been heavily criticized in the press (Pielke, 2016).

Increasingly sophisticated methods now allow WADA laboratories to detect drug levels lower than those detected by routine contamination analyses in pharmaceutical manufacturing. Even casual skin‐to‐skin contact or the use of cosmetic products may now produce positive doping tests (Brown, 2021a,2021a, 2021b). As a consequence, many positive test results are caused by unintentional internalization of banned substances in foodstuff, supplements, cosmetics or medicines, as was the case for Sachenbacher‐Stehle. One study claimed that more than 90% of the positive doping tests resulted from unintentional consumption of banned substances or the use of recreational drugs (Johannsen, 2018).

## Lack of decision limits

There are many doubtful cases where decision limits are at the core (Ordway & Verroken, 2021). Two recent cases affected Jarrion Lawson and Shelby Houlihan, two US track‐and‐field athletes who tested positive for small amounts of the anabolic steroids trenbolone (Lawson) and nandrolone (Houlihan). Both athletes appealed their suspensions to the Court of Arbitration for Sport (CAS), claiming that the positive tests were due to consumption of contaminated meat. Houlihan argued that the drug was from boar offal she had eaten the night before she gave her urine sample. Indeed, Emmanuel Strahm, a former scientist from the WADA laboratory in Stockholm, Sweden, confirmed that Houlihan’s test results “show all the evidence of boar meat or offal consumption the day prior to when the urine test was performed” (https://www.tas‐cas.org/fileadmin/user_upload/7977_Award__Reasoned__FINAL__for_publication.pdf). Lawson argued that he was first informed of the positive test two months after he had delivered his sample and that this long delay made it difficult to provide detailed information about how he was contaminated. Moreover, his defence team argued that the drug concentration was so low that it was impossible to discriminate it from accidental ingestion of contaminated food (https://www.doping.nl/media/kb/6463/CAS%202019_A_6313%20Jarrion%20Lawson%20vs%20IAAF%20%28OS%29.pdf; Gault, 2020).

Analyses of both Lawson’s and Houlihan’s hair demonstrated no long‐term exposure to steroids. Despite the similarities of the two cases, CAS concluded that Houlihan had committed an anti‐doping rule violation and upheld her suspension (https://www.tas‐cas.org/fileadmin/user_upload/7977_Award__Reasoned___FINAL__for_publication.pdf), but found it more likely that the steroids in Lawson’s urine was due to consumption of contaminated meat and annulled his suspension (https://www.doping.nl/media/kb/6463/ CAS%202019_A_6313%20Jarrion%20Lawson%20vs%20IAAF%20%28OS%29.pdf). The doping accusations and 18 months of suspension had, however, already damaged Lawson’s reputation and cost him considerable legal fees (Gault, 2020).

Even casual skin‐to‐skin contact or the use of cosmetic products may now produce positive doping tests.

## Accidental ingestion

Another similar case involved the Polish canoeist Adam Seroczynski who tested positive for clenbuterol during the 2008 Olympics in Beijing, China. Since clenbuterol was used in the production of meat in China and only a minute amount of the substance was detected in his urine, he argued that the clenbuterol came from food consumption. He also maintained that the amount detected would not have given him any performance‐enhancing effect. The International Olympic Committee (IOC) Disciplinary Commission nevertheless concluded in 2008 that Seroczynski had committed an anti‐doping rule violation, which he appealed to the CAS (https://www.doping.nl/media/kb/686/CAS%202009_A_1755%20Adam%20Seroczynski%20vs%20International%20Olympic%20Committee%20(IOC)%20(S).pdf).

At the CAS hearing, Seroczynski’s defence argued that the clenbuterol concentration in his urine was less than 0.4 ng/ml and much lower than the 2 ng/ml which WADA, in 2009, had set as the minimum concentration that its laboratories must be able to detect (https://www.doping.nl/media/kb/686/CAS%202009_A_1755%20Adam%20Seroczynski%20vs%20International%20Olympic%20Committee%20(IOC)%20(S).pdf). Other WADA laboratories might therefore – in accordance with the rules – have judged Seroczynski’s sample to be clean. The defence team also pointed out that there had been an epidemic of clenbuterol‐“poisoning” due to contaminated beef in Spain (1992), Italy (1996) and China (2006), and that scientific studies had shown that athletes who consume contaminated meat may test positive for anabolic agents. In one Spanish case, consumption of contaminated meat resulted in two persons with 2 and 4 ng clenbuterol per ml urine (https://www.doping.nl/media/kb/686/CAS%202009_A_1755%20Adam%20Seroczynski%20vs%20International%20Olympic%20Committee%20(IOC)%20(S).pdf).

In their response, the IOC pointed out that WADA’s regulations do not specify a minimum level of clenbuterol and that the presence of this substance, regardless of its concentration, constitutes a violation. Moreover, based on the principle of strict liability for athletes, the IOC claimed that the route by which clenbuterol came into Seroczynski’s body was not relevant (https://www.doping.nl/media/kb/686/CAS%202009_A_1755%20Adam%20Seroczynski%20vs%20International%20Olympic%20Committee%20(IOC)%20(S).pdf). The IOC also stated that an adverse analytical finding caused by clenbuterol‐contaminated food is extremely rare and unlikely to occur, especially because the organizing committee for the games had taken action prior to and during the games to prevent food contamination. Furthermore, they argued that Seroczynski was the only one to test positive for clenbuterol during the games and that there would have been others if contaminated food had been a problem (https://www.doping.nl/media/kb/686/CAS%202009_A_1755%20Adam%20Seroczynski%20vs%20International%20Olympic%20Committee%20(IOC)%20(S).pdf). CAS concluded that Seroczynski had committed an anti‐doping violation and upheld the decision issued by the IOC Disciplinary Commission (https://www.doping.nl/media/kb/686/CAS%202009_A_1755%20Adam%20Seroczynski%20vs%20International%20Olympic%20Committee%20(IOC)%20(S).pdf).

The case against Adam Seroczynski became a larger issue when a documentary by Hajo Seppelt from the German broadcaster ARD revealed that clenbuterol had also been found in samples from Jamaican sprinters and other, unnamed, athletes at the same Olympic Games in Beijing. Remarkably, the IOC – after having been made aware of this fact in 2016 – defended its lack of action against these athletes by refuting its own arguments presented at the Seroczynski hearing (Brown, 2017; Morgan, 2017): the case of the Jamaican sprinters was not revealed because both WADA and IOC now considered the concentration of clenbuterol to be low and without performance‐enhancing effects. The drug levels were all below 1 ng/ml and, in their opinion, most consistent with meat contamination (Brown, 2017; Morgan, 2017). Seroczynski’s urine sample contained less than 0.4 ng/ml clenbuterol. (https://www.doping.nl/media/kb/686/CAS%202009_A_1755%20Adam%20Seroczynski%20vs%20International%20Olympic%20Committee%20(IOC)%20(S).pdf). The inconsistency of IOC’s and WADA’s argumentation would justify a re‐examination of Seroczynski’s case.

There are also examples where laboratory analyses can themselves introduce contaminations.

## Sample contamination

There are also examples where laboratory analyses can themselves introduce contaminations. A prominent example is the case against the German medical student and amateur runner Benedikt Karus. By the use of gel electrophoresis, the WADA laboratory in Cologne identified rEPO in the A‐sample screening test of his urine. However, much, if not all, of the rEPO observed in Karus’ sample lane was leakage of rEPO from neighbouring control lanes. Interestingly, the WADA‐accredited laboratory in Tokyo, Japan, did not detect any rEPO when they independently tested the same sample using mass spectrometry. Karus was nevertheless sanctioned for 4 years (Nissen‐Meyer *et al*, 2019; https://web.archive.org/web/20180530151739/https://www.benedikt‐karus.de/).

By combining the principle of strict liability with anti‐doping tests without specified decision limits, WADA has chosen what is for them a trouble‐free procedure to identify dopers at the cost of punishing innocent athletes. A science‐based, more reliable and fairer approach would mandate the introduction of unambiguous decision limits for each drug. This may allow a few genuine cheats to remain undetected, but it is not likely to cause a higher prevalence of doping. The irony of today’s seemingly vigorous fight against doping is that the prevalence of doping in elite sports is still high – 30% or even higher according to a recent study (Ulrich *et al*, 2018) – and that only few cheating athletes are caught. Introducing science‐based decision limits would therefore make anti‐doping tests more trustworthy and strengthen the anti‐doping instrument as well as the rule of law for athletes.

## Supporting information



Review Process File
