# Supplementary material for: Are doping tests in sports trustworthy? Athletes suffer from insufficiently defined criteria for doping tests
Source: EMBO Rep. 2022 Feb 14;23(3):e54431. doi: 10.15252/embr.202154431 (PMC8892263; doi:10.15252/embr.202154431)
Supplement: Supplementary file 1 — Review Process File [file EMBR-23-e54431-s001.pdf]

EMBO reports

Are doping tests in sports trustworthy?

Jon Nissen-Meyer, Tore Skotland, and Erik Boye
DOI: 10.15252/embr.202154431

Corresponding author(s): Jon Nissen-Meyer (jon.nissen-meyer@ibv.uio.no)

Review Timeline:

Submission Date:	2nd Dec 21
Accepted:	1st Feb 22

Editor: Holger Breithaupt

Transaction Report:

This article was editorially reviewed.

(Note: With the exception of the correction of typographical or spelling errors that could be a source of ambiguity, letters and reports are not edited. Depending on transfer agreements, referee reports obtained elsewhere may or may not be included in this compilation. Referee reports are anonymous unless the Referee chooses to sign their reports.)
